# Effect of pH-Shifted Compound Heating Treatment on the Structure and Properties of Walnut Protein Isolate

**DOI:** 10.3390/foods14101754

**Published:** 2025-05-15

**Authors:** Liwen Chai, Wei Shi, Yunxia Tan, Xudong Che, Jiankang Lu, Bingyao Bai, Chunlan Zhang

**Affiliations:** 1 College of Food Science and Engineering, Tarim University, Alar 843300, China; clw15937457327@163.com (L.C.); shiwei18799900919@163.com (W.S.); tanyunxia98@163.com (Y.T.); 18291852975@163.com (X.C.); 12010034@taru.edu.cn (J.L.); 2 Production & Construction Group Key Laboratory of Special Agricultural Products Further Processing in Southern Xinjiang, Alar 843300, China

**Keywords:** walnut protein isolate, pH-shifted, walnut protein functionality, walnut protein structural properties

## Abstract

This study aims to explore the effect of pH on the solubility of walnut protein isolate (WPI) across a pH range of 7.0 to 12.0. The findings reveal that WPI solubility increased with rising pH levels, reaching a maximum solubility of 61.13% (4.79 mg/mL) at pH 12.0. Building on these results, WPI was subjected to compound heating at pH 12.0, with temperatures ranging from 60 °C to 100 °C (maintained for 30 min), to evaluate its structural and functional properties. Compared to the control group, WPI solubility peaked at 80.56% when heated to 90 °C. Additionally, its foaming capacity rose to 118.22% ± 7.34, accompanied by improved foaming stability. The average particle size decreased to 151.93 nm, while the surface charge increased to −28.33 mV. The protein subunits progressively aggregated within the range of 20.0 kDa to 14.1 kDa, and the surface hydrophobicity decreased. Scanning electron microscopy revealed that the surface morphology of the WPI became increasingly smooth with rising heating temperatures. Moreover, significant changes were observed in the secondary structure of the WPI. This study underscores the potential of pH-shifted compound heating treatment as a promising processing technique for WPI, offering key insights into the optimization of walnut protein processing.

## 1. Introduction

Protein solubility, which refers to the capacity of a protein to dissolve in an aqueous solution, serves as a key measure of its functionality. Highly soluble proteins demonstrate superior functional characteristics, including enhanced foaming and emulsifying properties [1]. Walnut protein isolate (WPI) content of glutelin, albumin, globulin, and prolamin were 72.06%, 7.54%, 15.67%, and 4.73% of the total extractable protein, respectively [2]. The solubility of WPI varies under different pH conditions. It exhibits lower solubility in neutral and mildly acidic environments but demonstrates significantly higher solubility in alkaline conditions [3], indicating its alkali-soluble nature.

Common modification methods employed in the food industry include ultrasound, high pressure, pH adjustment, and heat treatment. Among these, heat treatment has garnered significant attention due to its simplicity and cost-effectiveness [4,5,6,7,8]. This approach involves partially unfolding the protein structure through heating, which exposes internal sulfhydryl groups and hydrophobic side chains. Such exposure enhances interactions between protein molecules, thereby altering their structure and functionality. For instance, studies have demonstrated that heating at varying temperatures (40–100 °C) for durations of 10, 20, or 30 min significantly improves the solubility, foamability, and emulsification properties of gliadin [7]. Despite substantial research on heat treatment, the combined effects of alkaline conditions and heat on WPI remain inadequately studied. To address this gap, the present study examines the impact of pH-shifted compound heating treatment on WPI. Specifically, WPI is dissolved in a solution adjusted to a pH of 12.0 and subjected to heating at 60, 70, 80, 90, and 100 °C for 30 min. The structural changes are analyzed using polyacrylamide gel electrophoresis (SDS-PAGE), free sulfhydryl content (R-SH) analysis, endogenous fluorescence spectroscopy, surface hydrophobicity (H0) measurements, and scanning electron microscopy (SEM). These findings provide a theoretical basis for advancing WPI applications within the food industry.

## 2. Materials and Methods

### 2.1. Materials

Walnut meal sourced from Xinjiang Hetian Fruit Beginnings Foods Co. (Hotan, China) was defatted, and WPI (whey protein isolate) was prepared following the method described by Yan et al. [9]. The protein content was determined using the Kjeldahl method as specified in GB 5009.5-2016 [10]. The sample digestion was performed using an automatic Kjeldahl nitrogen detector, and the protein conversion factor for walnut protein was calculated to be 5.3, in accordance with the appendix of the national standard. The protein content was measured to be 80.35% ± 0.74. Other reagents used in the study included disodium hydrogen phosphate, sodium dihydrogen phosphate, and sodium chloride, all provided by Tianjin Zhiyuan Chemical Reagent Co. (Tianjin, China); a gel preparation kit from Beijing Lanjieke Technology Co. (Beijing, China); and a protein marker (range: 14.4–94.00 KDa) obtained from Tiangen Biochemical Technology (Beijing) Co. (Beijing, China). All the reagents utilized in the experiments were of analytical grade.

### 2.2. pH-Shifted Compound Heating Treatment of WPI

We weighed 1 g of WPI into a beaker and added 100 mL of phosphate buffer (PBS) with a concentration of 0.02 mol/L and a pH of 7.0. We stirred the mixture magnetically at room temperature for 2 h to prepare a 1% WPI solution. We allowed the solution to rest at 4 °C overnight to ensure complete protein hydration. This method was slightly modified from Zhao and Meng’s protocol [11]: the pH was adjusted to 12 ± 0.2 using 6 mol/L NaOH, followed by magnetic stirring for 2 h. Next, the solution was heated to temperatures of 60 °C, 70 °C, 80 °C, 90 °C, and 100 °C, respectively, maintaining each temperature for 30 min. Immediately afterward, the solution was cooled in an ice bath to obtain the treated protein solution, with an unheated sample serving as the control. The prepared pH-shifted compound heating-treated WPI was supplemented with 0.02% NaN3 and stored at 4 °C for subsequent analysis.

### 2.3. Determination of Solubility

The 1% WPI solution described above was centrifuged at 7000 rpm for 15 min, after which the protein content in the supernatant was measured using the Coomassie Brilliant Blue assay method [12].

The formula for solubility (%) is calculated as follows:

$$\mathrm{Solubility}\;\left(\%\right)=\frac{\mathrm{Protein}\;\mathrm{content}\;\mathrm{in}\;\mathrm{supernatant}}{\mathrm{Total}\;\mathrm{protein}\;\mathrm{content}\;\mathrm{in}\;\mathrm{sample}}\times 100$$

### 2.4. Determination of Foaming Capacity (FA) and Foaming Stability (FS)

Following a slightly modified version of the method described by Wang, 15 mL of 1% (*w*/*v*) WPI solution was transferred into a 50 mL test tube and stirred at a high speed of 10,000 rpm for 2 min. The total volume of the solution and foam in a measuring cylinder was then recorded as *V*_1._ After allowing the mixture to stand for 30 min, the total volume of the solution and foam was measured again, recorded as *V*_2_.

FA and FS were calculated using the following equations [13]:

$$\mathrm{FA}\;(\%)=\frac{V_{1}-15}{15}\times 100$$

$$\mathrm{FS}\;(\%)=\frac{V_{2}-15}{V_{1}-15}\;\times \;100$$

### 2.5. Determination of Particle Size and Potential

The WPI solution was diluted with PBS (0.02 mol/L, pH 8.0) to achieve a protein concentration of 0.1 mg/mL. The particle size and zeta potential were then measured using a Malvern particle sizer [14].

### 2.6. Determination of SDS-PAGE

We used Jardine’s method with appropriate modifications [15]. The separating gel had a concentration of 12%, while the concentrating gel’s concentration was set at 5%. The WPI solution, adjusted to a concentration of 1 mg/mL, was mixed with spiking buffer composed of 0.25 M Tris-HCl, 10% SDS, 0.5% bromophenol blue, 50% glycerol, and 5% β-mercaptoethanol at a ratio of 4:1, resulting in a final concentration of 0.8 mg/mL. The prepared mixture was boiled for 5 min at 100 °C and then allowed to cool to room temperature. Subsequently, 20 μL of the mixture and 10 μL of the standard protein were loaded into gel wells. Electrophoresis was conducted with voltages set at 40 V and 120 V, respectively. Following electrophoresis, the gel was stained for 30 min using Coomassie Brilliant Blue R-250 and then repeatedly decolorized with a solution of glacial acetic acid and methanol.

### 2.7. Determination of Free Sulfhydryl Content (R-SH)

The concentration of R-SH in the WPI solution was measured using the Ellman method [16]. For the determination of R-SH, 1 mL of the sample solution was mixed with 5 mL of Tris-Gly buffer solution, followed by the addition of 0.1 mL of Ellman’s reagent. The mixture was then incubated at 25 °C for 15 min and its absorbance was measured at 412 nm. A solution without Ellman’s reagent was used as the blank control.

R-SH content (μmoL/g) = (73.53 × A412 × D)/C

73.53 = 106/13,600,

where 13,600 represents the molar extinction coefficient of Ellman’s reagent, D is the dilution factor, and C denotes the protein concentration in mg/mL.

### 2.8. Determination of Ultraviolet Absorption Spectra (UV)

The absorbance of the sample was measured following the method outlined by Li et al. [17]. The WPI solution was dissolved in PBS (pH 7.0, 0.02 mol/L) to achieve a final protein concentration of 0.1 mg/mL. The UV absorption spectra of the proteins were recorded using a UV spectrophotometer, with measurements conducted across a wavelength range of 200–360 nm and a slit width of 2 nm. Each sample set was scanned three times, and the dataset displaying the most consistent peak variations was selected for analysis.

### 2.9. Determination of Endogenous Fluorescence Spectra

The WPI solution was diluted to a concentration of 0.1 mg/mL using PBS (0.02 M, pH 8.0), and the fluorescence spectra of the proteins were analyzed with a fluorescence spectrophotometer. The instrument parameters were set as follows: a slit width of 5 nm, a scanning speed of 1200 nm/min, an excitation wavelength of 290 nm, a scanning range of 300–400 nm, a scanning interval of 20 ms, and a reaction time of 0.1 s [18].

### 2.10. Determination of H_0_

The H0 values of the five groups of protein solutions were determined using the 1-anilinaphthalene-8-sulfonic acid (ANS) method [19]. The WPI solution was diluted with PBS (0.02 M, pH 8.0) to achieve final concentrations of 0.05, 0.1, 0.15, 0.20, and 0.25 mg/mL. Two milliliters of the WPI solution were mixed thoroughly with 10 μL of an 8 mM ANS solution and then allowed to stand for 15 min in the dark. The fluorescence intensity of the mixture was measured using a fluorescence spectrophotometer at an excitation wavelength of 390 nm and an emission wavelength of 470 nm. The H0 value of the WPI solution was calculated based on the slope of the curve plotting fluorescence intensity against protein concentration.

### 2.11. Determination of SEM

Following the approach detailed by Jia et al., the prepared protein solution was freeze-dried, and the resulting powder was evenly distributed onto specialized conductive double-sided adhesive. The sample was then mounted on the coating stage for gold sputtering before being placed into a scanning electron microscope. The optimal field of view and magnification were adjusted to capture images and observe the microstructure [20].

### 2.12. Determination of FTIR

The methods for the determination of FTIR were as follows. Referring to Yang et al., the dried samples were thoroughly mixed with KBr in a 1:100 ratio, ground into a fine powder, and pressed into thin slices. These samples were then analyzed using a Fourier Transform Infrared Spectrometer. The scanning parameters were set to a range of 4000–400 cm^−1^, with a resolution of 4 cm^−1^, and 32 scans were performed [21].

### 2.13. Statistical Analysis

The data obtained were the values of three parallel determinations for each sample and the mean and standard deviation were calculated. Significance analyses were performed using SPSS 26.0 (*p* < 0.05 was considered significantly different) and plots were made using Origin 2021.

## 3. Results

### 3.1. Influence of pH-Shifted Compound Heating Treatment of WPI on Solubility

**Figure 1:**
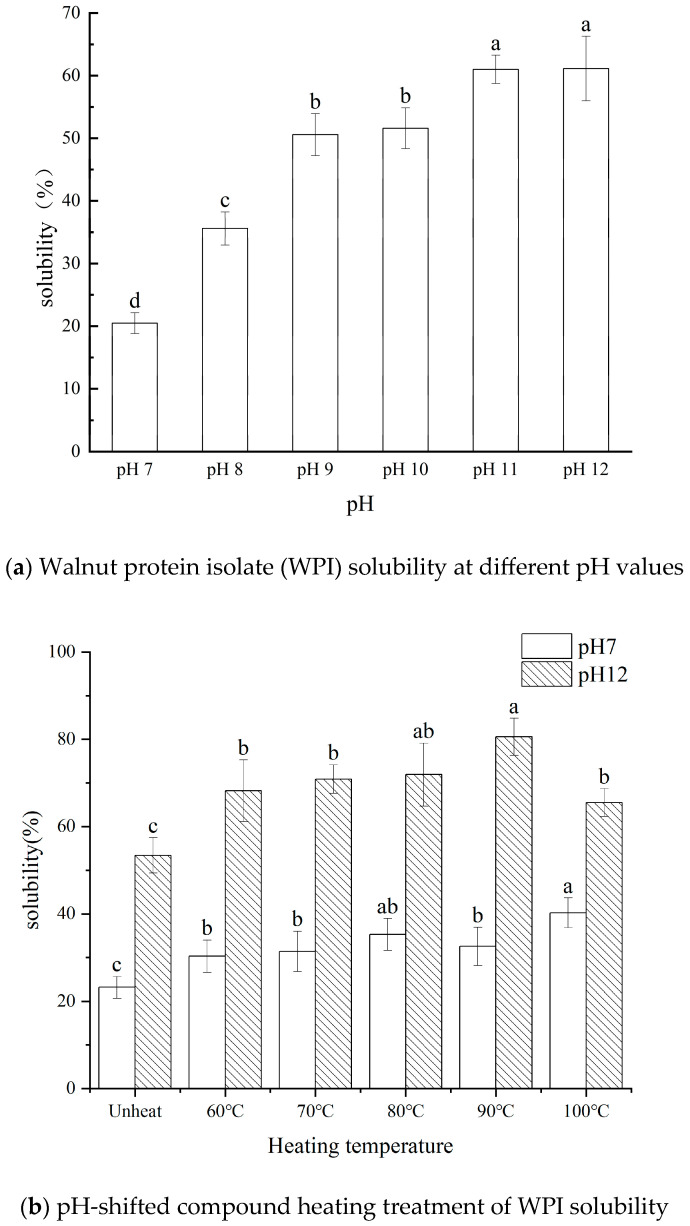
WPI solubility at different pH values, a–d indicates the significant differences between different pH (*p* < 0.05) (**a**) and pH-shifted compound heating treatment WPI solubility a–c indicates the significant differences between different temperature (*p* < 0.05) (**b**).

Optimal solubility plays a crucial role in improving protein production and processing efficiency [22]. In Figure 1a, the solubility of WPI increases significantly (*p* < 0.05) following various pH-shifted treatments. The lowest solubility, recorded as 20.47% ± 1.66 (1.61 mg/mL), occurs at pH 7.0. However, solubility progressively improves with increasing pH, peaking at 61.13% (4.79 mg/mL) at pH 12.0 [23]. This trend can likely be attributed to the hydrolysis of WPI under alkaline conditions. Moreover, the cleavage of amide bonds in WPI at higher pH levels may transform these groups into carboxyl groups (-COOH), thereby increasing the protein’s negative charge and electrostatic repulsion. This transformation weakens hydrogen bonding and van der Waals forces within the protein, promoting structural unfolding and enhancing hydration capacity [24].

Under alkaline shift treatment, the WPI solutions displayed increasingly darker coloration alongside reduced precipitation. Notably, at pH 11.0 and pH 12.0, no significant precipitation occurred. This observation suggests that alkaline conditions may lead to alter protein structures, and strengthen protein–water interactions [25]. Alkaline shift treatment has proven to be an effective approach for improving WPI solubility, which is why pH 12.0 was selected for the subsequent complex heating process of the WPI.

Previous studies have demonstrated that while alkali or heat treatments alone can alter protein structure, their individual effects on solubility are negligible [26]. Wang et al. investigated pea proteins and observed that untreated controls, alkali-treated samples, and samples subjected to an alkaline–thermal compound treatment at pH 12.0 and 60 °C exhibited varying levels of solubility. The highest solubility (88.49%) was achieved with the alkaline–thermal compound treatment, which modified the protein structure, reduced the particle size, and increased the surface charge. These structural changes are considered the primary factors contributing to the enhanced solubility of pea proteins. The findings suggest that while individual alkali or heat treatments produce limited effects, the combined alkaline–thermal treatment significantly improves protein solubility [27]. Based on this research, we adopted a pH-shifted compound heating treatment for WPI to enhance its solubility and broaden its potential applications in the food industry.

In Figure 1b, the WPI at pH 12.0 underwent heat treatment in a water bath at temperatures of 60 °C, 70 °C, 80 °C, 90 °C, and 100 °C for 30 min. Compared to the control group at pH 7.0, the solubility of the WPI at pH 12.0 significantly improved after the heat treatment (*p* < 0.05), demonstrating that alkaline heat treatment effectively enhances WPI solubility. Moreover, as the temperature increased, the solubility of the WPI at pH 12.0 exhibited a consistent and noteworthy improvement (*p* < 0.05). Specifically, the solubility rose from 53.40% ± 4.03% (untreated) to 80.56% ± 3.19% at 90 °C. This enhancement can be attributed to the effects of the alkaline heat treatment, which promotes the unfolding of the protein’s molecular structure. This unfolding exposes internal hydrophobic groups, improving protein–water interactions and thereby significantly increasing solubility. However, excessively high temperatures can lead to heightened protein denaturation, causing reaggregation of the unfolded molecules and ultimately resulting in reduced solubility.

### 3.2. Effect of pH-Shifted Compound Heating Treatment of WPI on FA and FS

The foamability of proteins refers to their ability to generate foam through physical methods such as high-speed agitation or shaking, while foam stability denotes the capacity of protein-based foam to retain its structure over time. These attributes are essential functional characteristics of proteins and are strongly linked to their solubility [28].

**Figure 2:**
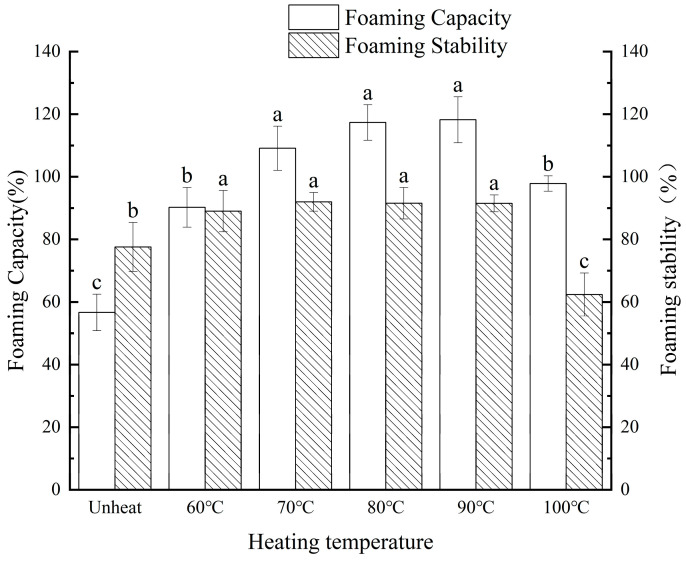
Effect of pH-shifted compound heating treatment on foaming property and foaming stability of walnut protein. a–c indicates the significant differences between different temperature (*p* < 0.05).

In Figure 2, the FA of the WPI showed a significant increase (*p* < 0.05) as the heating temperature rose, compared to the control group. Specifically, the FA of the unheated WPI gradually increased from 56.67% ± 5.81 to a peak of 118.22% ± 7.34 when heated at 90 °C for 30 min, after which it began to decline. Similarly, the FS exhibited a marked improvement (*p* < 0.05), reaching its maximum value of 91.49% ± 2.69 at 90 °C before decreasing. This behavior can be attributed to the unfolding of the protein structure at higher temperatures, which exposes hydrophilic groups, thereby enhancing foam formation and stability. However, excessive heating can lead to the formation of larger soluble aggregates, diminishing the flexibility of protein molecules and ultimately causing a decline in both foaming ability and foam stability [28].

### 3.3. Effect of pH-Shifted Compound Heating Treatment of WPI on Particle Size and Potential

**Figure 3:**
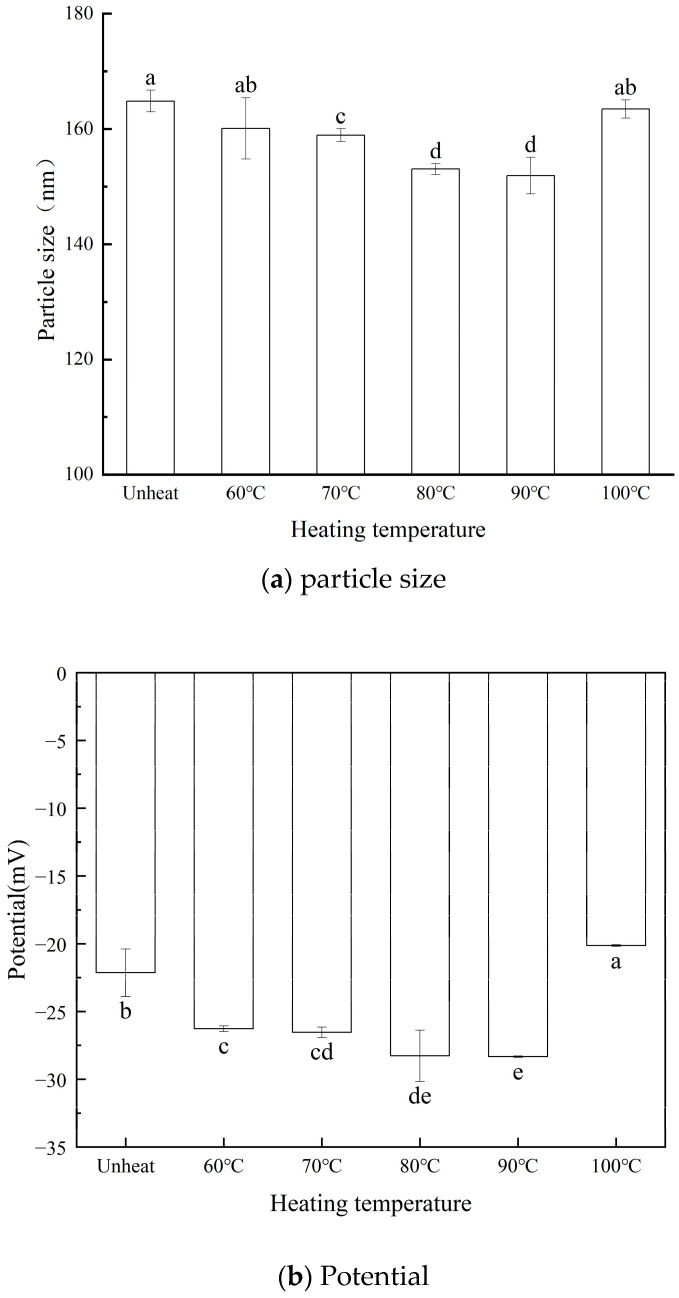
Effect of pH-shifted compound heating treatment on particle size, a–d indicates the significant differences between different temperature (*p* < 0.05) (**a**) and potential of WPI, a–e indicates the significant differences between different temperature (*p* < 0.05) (**b**).

In Figure 3a, the particle size of the pH-shifted WPI compound significantly decreased with increasing heating treatment (*p* < 0.05). Specifically, the protein solution’s particle size reduced from 164.87 ± 1.90 nm (untreated) to 151.93 ± 3.19 nm after 30 min of treatment at 90 °C, followed by an increase at higher temperatures. This behavior can likely be attributed to the formation of a more compact structure within the protein molecules, induced by the pH-shifted compound heating treatment of the WPI.

In Figure 3b, the negative surface charge of the composite-treated WPI increases with rising heating temperatures, as demonstrated by the absolute value of the zeta potential (*p* < 0.05). The zeta potential peaks at −28.33 ± 0.05 mV when the WPI is heated to 90 °C. This phenomenon can be attributed to the pH-adjusted compound heating treatment, which promotes the unfolding of protein molecules and exposes a greater number of charged amino acid residues. The enhanced electrostatic repulsion between the molecules surpasses the intermolecular attractive forces, thereby effectively preventing protein aggregation and ensuring a more stable solution distribution. However, at excessively high temperatures, the protein molecules undergo refolding, resulting in larger particle sizes and a diminished surface charge [29].

### 3.4. Effect of pH-Shifted Compound Heating Treatment of WPI on SDS-PAGE

**Figure 4:**
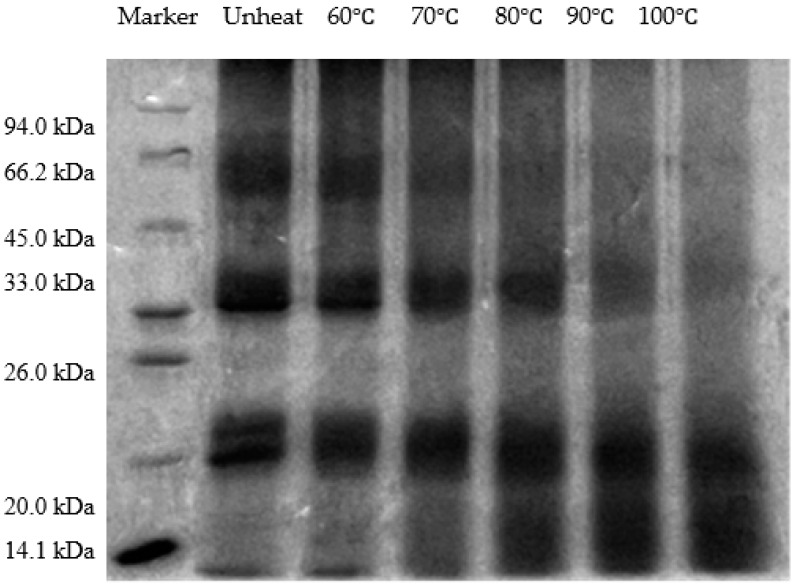
SDS-PAGE of WPI treated with pH-shifted compound heating treatment.

To examine the effects of the pH-shifted compound heating treatment on the structural modifications of the WPI, the degree of polymerization and dispersion were assessed using sodium dodecyl sulfate polyacrylamide gel electrophoresis (SDS-PAGE). As shown in Figure 4, the electrophoretic profile distinctly displayed bands corresponding to each subunit of the WPI. The findings indicated that the application of the pH-shifted compound heating treatment triggered significant alterations in the protein subunits. These changes included a progressive reduction in band intensity, likely due to the degradation of peptide chains within the WPI subunits under strongly alkaline conditions [30]. This observation aligned with increased solubility and decreased particle size, suggesting a relationship between the structural modifications and the functional properties of the treated proteins.

### 3.5. Effect of pH-Shifted Compound Heating Treatment of WPI on R-SH Content

R-SH influences the spatial conformational stability of proteins and impacts their functional properties [31].

**Figure 5:**
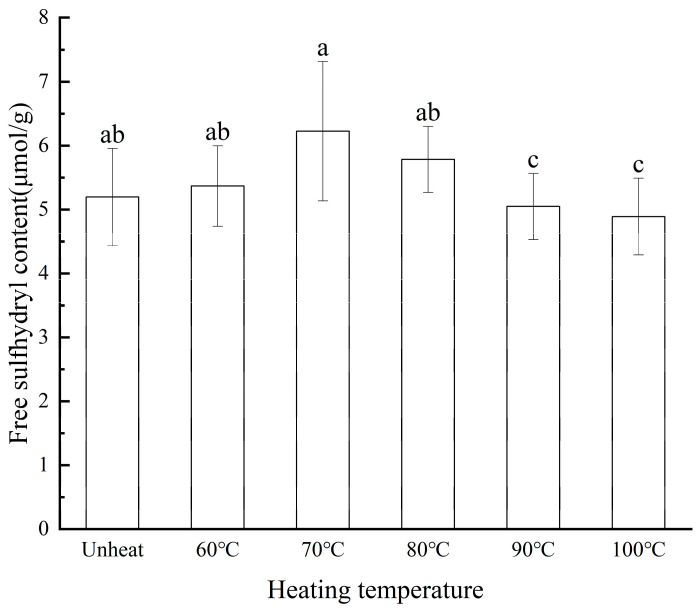
Effect of pH-shifted compound heating treatment on the R-SH of WPI, a–c indicates the significant differences between different temperature (*p* < 0.05).

In Figure 5, the R-SH content of the WPI subjected to the pH-shifted compound heating treatment remained relatively stable across the studied temperature range, with values ranging from 5.19 to 6.23 μmol/g. At lower heating temperatures (60–70 °C), partial protein denaturation occurs, leading to the unfolding of molecular structures and exposing previously buried free sulfhydryl groups (-SH), thereby increasing the free sulfhydryl content. As the temperature rises above 80 °C, complete protein denaturation takes place, further exposing free sulfhydryl groups. However, the simultaneous oxidation of these -SH groups results in the formation of disulfide bonds (-S-S-), causing a reduction in sulfhydryl content. At 100 °C, the sulfhydryl content may decline further, potentially due to protein aggregation, which buries the -SH groups within the molecular structure [32].

### 3.6. Effect of pH-Shifted Compound Heating Treatment of WPI on UV

**Figure 6:**
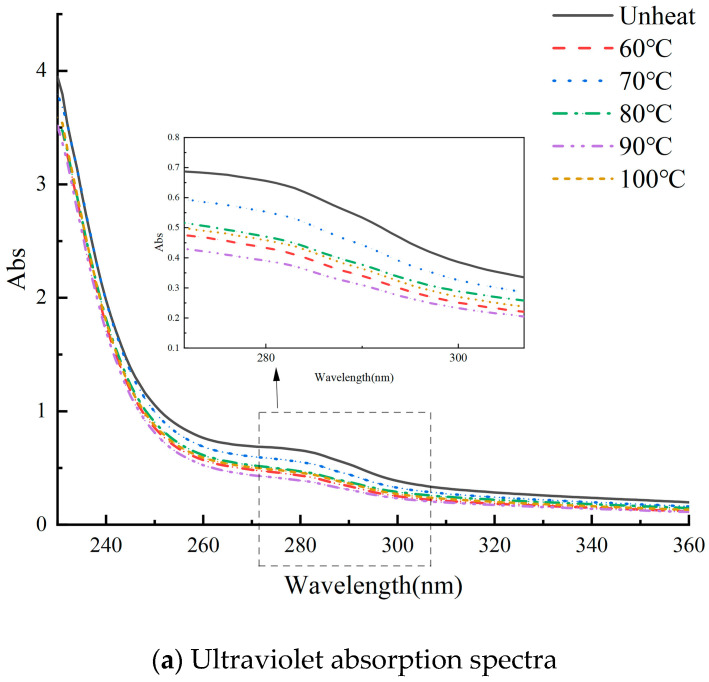

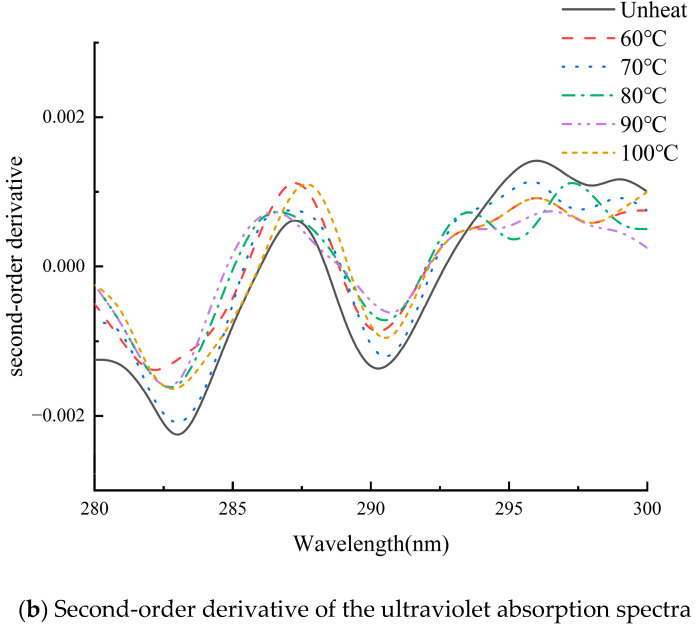
UV of WPI treated with pH-shifted compound heating treatment.

The chromogenic groups of three aromatic amino acids—tyrosine (Tyr), tryptophan (Trp), and phenylalanine (Phe)—exhibit distinct UV characteristics [17]. As shown in Figure 6a, the conventional UV spectral analysis method failed to adequately resolve their characteristic absorption peaks. In contrast, the second-order derivative profile offered a more precise representation of these peaks. Figure 6b illustrates the effects of the pH-shifted compound heating treatment in conjunction with the second-order derivative profile on the UV spectrum of the WPI.

In Figure 6b, distinct absorption peaks are observed within the wavelength range of 280 to 300 nm. Notably, the peak near 282 nm is attributed to Tyr residues [6,33], while the peak near 296 nm is associated with Trp residues [34,35]. The UV spectrum of the WPI subjected to the pH-shifted compound heating treatment displays characteristic absorption peaks near 282 nm, with their intensities ranked in descending order. In contrast, the characteristic absorption peaks near 296 nm follow a distinct order: unheated > 70 °C > 60 °C > 100 °C > 90 °C > 80 °C. This pattern reveals that the Trp content is consistently higher in the unheated WPI compared to the heated samples [36]. The opposing trends observed in the characteristic absorption peaks at 282 nm and 296 nm suggest that the tertiary structure of WPI undergoes partial unfolding under extreme pH and heat conditions, resulting in the exposure of amino acid residues [37]. However, excessively high temperatures may lead to protein aggregation, potentially affecting the spectral characteristics. The pH-shifted compound heating treatment influences the structure of the walnut protein isolate by modifying its protein conformation and altering the microenvironment surrounding the amino acid residues.

### 3.7. Effect of pH-Shifted Compound Heating Treatment of WPI on Endogenous Fluorescence Spectra

**Figure 7:**
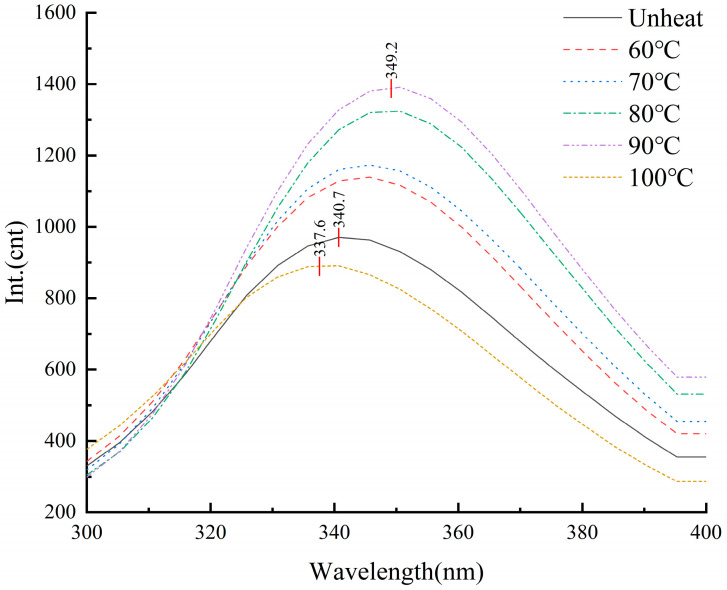
Endogenous fluorescence spectra of WPI after pH-shifted compound heating treatment.

The aromatic groups in protein side chains allow them to emit fluorescence when excited by 290 nm light, making fluorescence a valuable tool for monitoring changes in protein tertiary structure [38]. As illustrated in Figure 7, the maximum absorption wavelength (λmax) of the endogenous fluorescence in the pH-shifted compound heating treatment WPI ranged from 337.6 to 349.2 nm. In contrast, the unheated WPI exhibited a λmax of 340.7 nm. With increasing temperature, the fluorescence intensity rose, accompanied by a slight red shift in λmax, reaching 349.2 nm at 90 °C. However, at 100 °C, the fluorescence intensity declined, and a blue shift in λmax to 337.6 nm was observed, alongside a fluorescence burst reaction [39]. These results indicate that the pH-shifted compound heating treatment caused structural changes in the tertiary structure of the WPI. This conclusion aligns with the findings from the solubility, particle size, and SDS-PAGE analyses.

### 3.8. Effect of pH-Shifted Compound Heating Treatment of WPI on H_0_

The H_0_ of proteins is determined by the distribution of hydrophobic amino acids on their surface, as well as by the unfolding and deformation of their structure. It represents not only a surface property but also a vital indicator of the tertiary structural conformation of proteins, which is intimately tied to their structural and functional properties [40].

**Figure 8:**
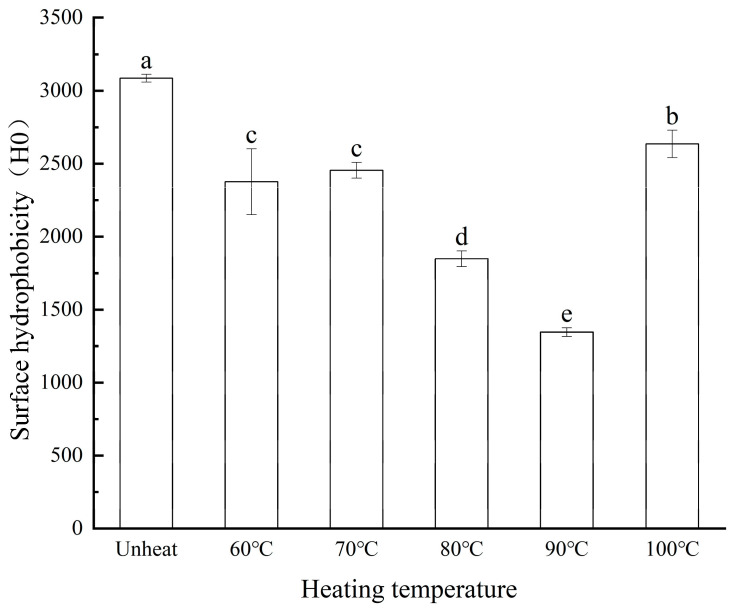
H_0_ of WPI treated by pH-shifted compound heating treatment, a–e indicates the significant differences between different temperature (*p* < 0.05).

The H_0_ of the WPI subjected to the compound pH-shifted compound heating treatment is presented in Figure 8. As the heating temperature increased, the H_0_ decreased significantly, from 3086.06 to 1346.4, when compared with the unheated control group (90 °C, 30 min). This phenomenon can be attributed to the negative correlation between H_0_ and solubility, particularly when the pH deviates from the protein’s isoelectric point [41]. The compound heating treatment following a pH shift induced varying degrees of structural changes in the WPI, leading to interactions between the protein molecules that directly affect H_0_. Under highly alkaline conditions, more hydrophobic groups are exposed in the WPI than under neutral conditions. However, during the pH-shifted compound heating treatment process, the hydrophobic groups reach a threshold, triggering hydrophobic collapse. This collapse causes protein molecules to aggregate through the hydrophobic interactions, ultimately reducing the H_0_ [42].

### 3.9. Scanning Electron Microscopy of pH-Shifted Compound Heating Treatment WPI

SEM is widely employed in food science due to its exceptional resolution and ability to observe and analyze the microstructural morphology of samples [43].

**Figure 9:**
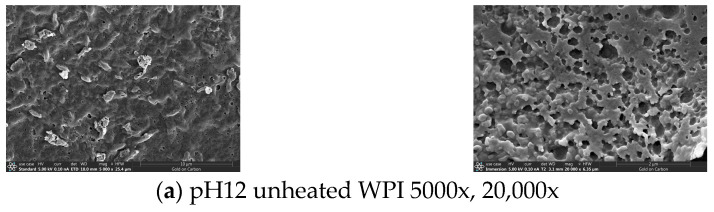

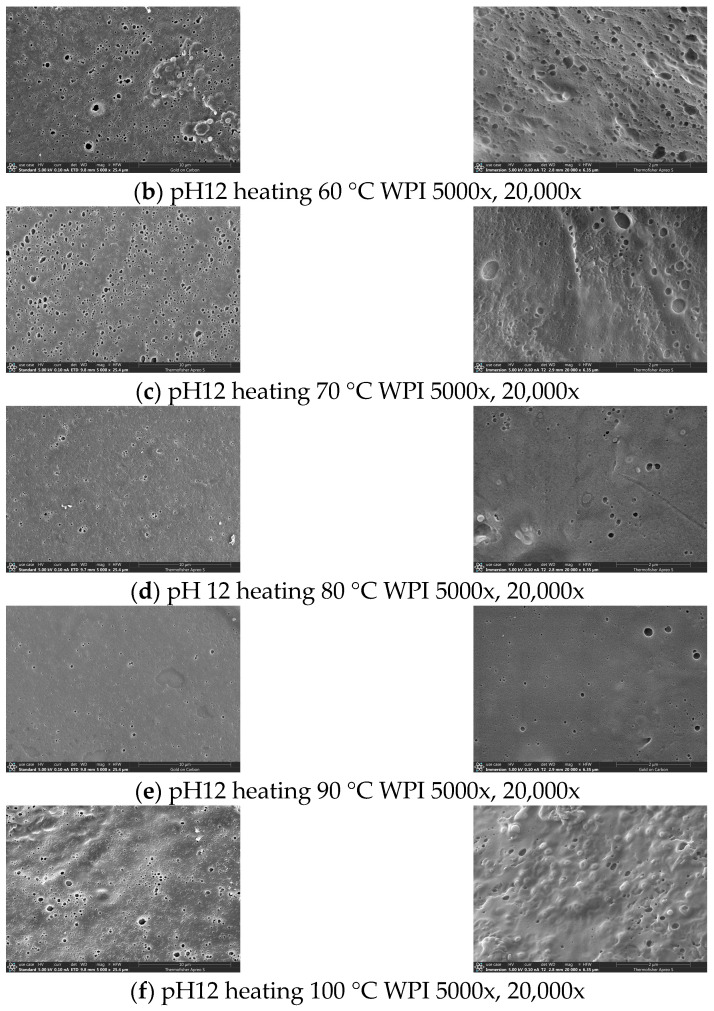
SEM of WPI treated with pH-shifted compound heating treatment.

Figure 9 displays the SEM images of the WPI subjected to the compound pH-shifted heating treatment. Figure 9a depicts the surface morphology of the unheated WPI powder, characterized by an irregular surface with attached fragments and a combination of dense and sparse pores. The micrographs (b) through (f) illustrate the impact of progressively increasing the heating temperatures. As the temperature rises, the surface of the WPI powder becomes increasingly smooth, with the surface attachments disappearing entirely at 90 °C. Simultaneously, most air transport pores shrink and eventually vanish. This phenomenon may result from high-temperature treatment causing proteins to unfold, exposing hydrophobic groups and strengthening intermolecular interactions—such as hydrophobic aggregation and disulfide bond recombination—which lead to the contraction of the protein network and the filling of the original pores. When the heating temperature reached 100 °C, uneven air vents increased, and bulges appeared on the surface in Figure 9f. This phenomenon is likely attributed to the formation of larger aggregates from soluble aggregates, consistent with the findings from the particle size analysis and SDS-PAGE results.

### 3.10. Effect of pH-Shifted Compound Heating Treatment of WPI on FTIR

The secondary structure of the WPI was analyzed via FTIR. The protein displayed distinct absorption bands: the amide I band (1600–1700 cm^−1^), corresponding to C=O stretching vibrations; the amide II band (1525–1550 cm^−1^), associated with -NH_2_ bending and C-N stretching vibrations; and the amide III band (1230–1350 cm^−1^), attributed to C-N vibrations [44,45].

**Figure 10:**
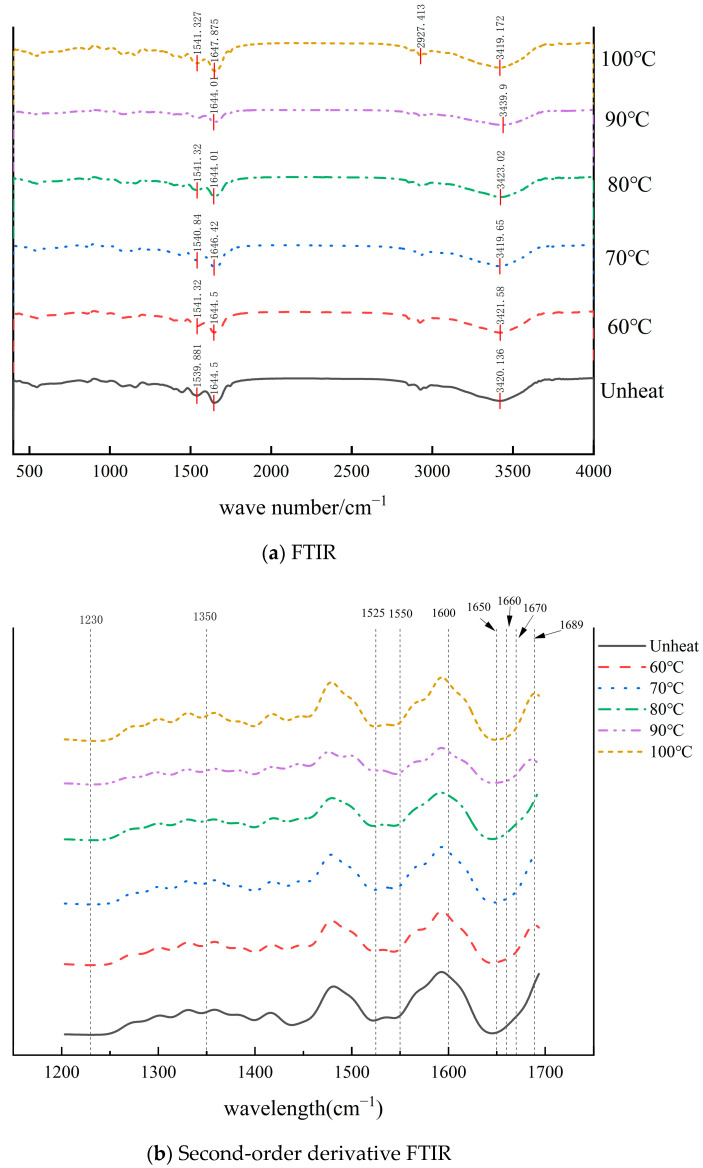
FTIR of pH-shifted compound heating treatment with WPI.

In Figure 10a, the WPI exhibited two distinct absorption peaks at approximately 1644 cm^−1^ and 1540 cm^−1^, corresponding to the amide I and amide II bands, respectively. The broad absorption peak observed at 3425 cm^−1^ is likely attributable to the stretching vibrations of hydroxyl groups (-OH) or the presence of extensive intramolecular or intermolecular hydrogen bonds within the protein aggregates [46]. Notably, the peak position shifted from 3420.13 cm^−1^ (unheated) to 3439.9 cm^−1^ at 90 °C, followed by a decrease to 3419.17 cm^−1^ at 100 °C. This shifting behavior indicates that intramolecular hydrogen bonding within the protein structure was disrupted during the pH-shifted compound heating treatment [47].

To further investigate the changes in the secondary structure, the original FTIR spectra (1200–1700 cm^−1^) underwent second-order derivative analysis, as shown in Figure 10b. The analysis revealed no notable variations in the amide III band (1230–1350 cm^−1^). However, within the amide I band (1600–1700 cm^−1^), the absorption peaks corresponding to the α-helix structure (1650–1660 cm^−1^) broadened as the temperature increased. In contrast, the β-sheet absorption peaks (1670–1689 cm^−1^) exhibited significant changes, with the β-sheet content initially increasing before subsequently decreasing. These findings suggest that the pH-shifted heating treatment caused substantial alterations in the secondary structure of the WPI.

## 4. Conclusions

In summary, after the application of the pH-shifted compound heating treatment to the WPI, the intensity of the SDS-PAGE subunits decreases, the free sulfhydryl content changes, the fluorescence intensity increases accompanied by a blue shift, and scanning electron microscopy reveals a reduction in pores. Additionally, the infrared spectral peak shifts from 3420.13 cm^−1^ (unheated) to 3439.9 cm^−1^, indicating the disruption of the intramolecular hydrogen bonds within the protein structure. These changes reflect alterations in the primary, secondary, and tertiary structures of the walnut protein isolate. The structural modifications resulted in a reduced particle size, increased surface charge, and lower molecular weight. Compared to high-speed shearing treatment under alkaline conditions, the pH-shifted compound heating treatment increased the solubility of the WPI from 76.65% to 80.56%, while also significantly improving its FA and FS. The findings highlight that the pH-shifted compound heating treatment serves as an effective method for WPI modification, demonstrating strong application potential in plant-based beverages and low-viscosity protein solutions.

## Data Availability

The original contributions presented in the study are included in the article. Further inquiries can be directed to the corresponding authors.
